# SSRIs treatment did not completely restore affective state in patients with the initial clinically confirmed major depressive disorder/generalized anxiety disorder after COVID-19 disease

**DOI:** 10.1192/j.eurpsy.2022.279

**Published:** 2022-09-01

**Authors:** J. Fedotova, Z. Bereza

**Affiliations:** 1 I. P. Pavlov Institute of Physiology RASci, Department Of Neuroendocrinology, St. Petersburg, Russian Federation; 2 Medical Center “Bekhterev”, Psychiatry, St. Petersburg, Russian Federation

**Keywords:** Covid-19, depression, anxiety, SSRIs, pharmacotherapy

## Abstract

**Introduction:**

The major clinical outcomes of COVID-19 in the brain are associated with its deleterious neurological and mental health actions.

Today, there are limited findings concerning the studying of neuropsychiatric action for SARS-Cov-2 in humans after COVID-19 disease.

**Objectives:**

The aim of the present study was to compare the efficacy of SSRIs (escitalopram, sertraline and fluoxetine) for 6 months therapy on the affective profile of man and women with the clinically confirmed Major Depressive Disorder (MDD) or Generalized Anxiety Disorder (GAD) cases following COVID-19 disease.

**Methods:**

. For the assessment of affective profile in man and women (30-55 years) with the initial clinically confirmed MDD or GAD cases after COVID-19 disease, we used the different tests: Montgomery-Asberg Depression Rating Scale (MADRS) and anxiety scale (ShARS Scale). The hormonal and monoamines levels in the serum blood were measured by ELISA tests before and after SSRIs therapy.

**Results:**

After 6 months of SSRIs therapy, MADRS Scale showed a incomplete disappearance of the depressive/anxiety manifestations in both men and women with the initial clinically confirmed MDD case after COVID-19 (p<0,05). We found that SSRIs were able to reduce depression/anxiety levels only on 20% in man or on 30% in women with the initial MDD case after COVID-19 before treatment.

**Conclusions:**

SSRIs treatmet alone failed to produce the decrease of depression/anxiety in the patients of both gender with the initial MDD or GAD diagnosis after COVID-19. The further randomized clinical trials involving new pharmacological therapies for psychiatric pations after COVID-19 disease are needed.

**Disclosure:**

No significant relationships.

